# Attention-Enhanced Autoencoder with Marginal-Variance-Regularized Feature Reconstruction for Imbalanced Insurance Policy-Ownership Classification

**DOI:** 10.3390/e28090985

**Published:** 2026-09-03

**Authors:** Jiaming Tian, Qingyi Ding, Bohan Li, Xiao Yang

**Affiliations:** 1 School of Business, Soochow University, Suzhou 215222, China; 2312503067@stu.suda.edu.cn (J.T.); 2312363043@stu.suda.edu.cn (Q.D.); 2428362043@stu.suda.edu.cn (B.L.); 2 Department of Electrical Engineering & Computer Science, University of Wyoming, 1000 E. University Ave., Laramie, WY 82071, USA

**Keywords:** policy-ownership classification, class imbalance, insurance tabular data, autoencoder, marginal-variance regularization, channel attention, feature reconstruction

## Abstract

Identifying the small group of customers who hold a given policy in severely imbalanced tabular data is a recurring screening problem in insurance analytics. This study considers binary caravan-insurance policy-ownership classification on the COIL 2000 benchmark, where the positive-class prevalence is below 6%. The benchmark is a single cross-section, so the label describes current ownership rather than a future purchase event. We propose an Attention-based Symmetric AutoEncoder (ASAE) that combines an auxiliary symmetric reconstruction branch, a channel attention gate, and a marginal log-variance regularizer on a 32-dimensional latent representation. The regularizer operates on individual latent variances and is treated as a heuristic rather than as an estimator of joint differential entropy. Under a common tuning and evaluation protocol on a stratified partition, the ASAE is compared with five conventional machine learning methods and seven neural models. Across five paired runs, it achieves an F1-score of 0.6008 ± 0.0115 and an area under the receiver operating characteristic curve (AUC) of 0.8584 ± 0.0034. Relative to TabNet, the strongest baseline considered, the mean differences are 0.064 in F1-score (95% confidence interval 0.043–0.085) and 0.032 in AUC (95% confidence interval 0.017–0.048). The ordering is preserved across five stratified re-splits, four imbalance-handling configurations, and a complete type-consistent preprocessing rerun in which nominal attributes are one-hot encoded, oversampled with SMOTENC, and reconstructed with categorical cross-entropy losses (F1-score 0.6241 ± 0.0074, AUC 0.8702 ± 0.0050). All reported results use stratified random partitions of COIL 2000. Because 27% of the records share an identical predictor vector with another record, the official challenge separation and two grouped partitions are also defined, so that exact-duplicate and sociodemographic overlap can be isolated from the primary split. The training code, split indices, and per-run predictions used for the reported tables are publicly available.

## 1. Introduction

Insurance analytics frequently requires models that recognize a small group of policyholders among a much larger population that does not hold the policy in question. The COIL 2000 benchmark provides a compact instance of this setting: its target column records whether a customer currently holds a caravan (mobile home) insurance policy, with fewer than 6% of the observations belonging to the positive class [1,2]. The dataset is a single cross-section without acquisition dates or policy-inception timestamps, so the label describes ownership status at the time of collection rather than a future purchase event. We therefore treat the task throughout as policy-ownership classification. A campaign-targeting reading would require longitudinal data and is not examined here. The resulting imbalance makes overall accuracy uninformative and places greater emphasis on ranking quality and the precision–recall trade-off.

Conventional customer-scoring models, including logistic regression and tree ensembles, provide useful baselines but may require substantial feature engineering to represent nonlinear interactions among demographic and product-ownership variables. COIL 2000 contains 85 mixed-type predictors and one target column, so it offers a compact but challenging test of representation learning under severe class imbalance. The benchmark also contains substantial record duplication—27.1% of the 9822 records share an identical 85-dimensional predictor vector with at least one other record—so the partitioning protocol is part of the experimental design.

Gradient-boosted tree ensembles such as XGBoost and LightGBM model nonlinear tabular relationships effectively [3,4]. Neural representation-learning methods offer a complementary approach by learning a shared latent representation that can be optimized jointly with the classification objective. Their performance on modest tabular datasets, however, depends strongly on architecture, regularization, and model-selection protocol [5,6].

Autoencoders map high-dimensional inputs to a compact bottleneck and learn to reconstruct the inputs [7]. Denoising, contractive, and variational variants impose different regularization structures on the learned representation [8,9,10]. In supervised settings, reconstruction can also serve as an auxiliary training objective, although the presence of decoder skip connections means that reconstruction error alone cannot establish what information is retained in the bottleneck.

Two design questions motivate the present model. First, an auxiliary reconstruction objective may regularize a supervised encoder, but its contribution should be assessed empirically rather than inferred from reconstruction error. Second, hidden dimensions can differ in their usefulness for ownership classification. Channel attention, adapted from squeeze-and-excitation networks [11], provides a learnable mechanism for reweighting these hidden activations.

This paper proposes the Attention-based Symmetric AutoEncoder (ASAE). The model combines a symmetric auxiliary reconstruction branch, a channel attention gate after the first encoder layer, and a marginal log-variance regularizer on the bottleneck. The regularizer increases the average log variance of individual latent dimensions but is not treated as an estimator of joint differential entropy. The contributions are as follows: (1) a jointly trained reconstruction and marginal-variance regularization objective; (2) a channel attention module adapted to a fully connected tabular encoder; and (3) a controlled comparison with twelve classical and neural baselines, supported by component ablations, paired statistics, covariance-spectrum diagnostics, and robustness analyses across data splits and imbalance-handling configurations. Official and grouped partitions are also defined so that exact-duplicate and sociodemographic overlap can be isolated from the primary stratified split.

The remainder of the paper reviews insurance policy-ownership classification and tabular representation learning, describes the dataset and ASAE methodology, reports the comparative and robustness results, and discusses the scope and limitations of the findings.

### 1.1. Related Work

#### 1.1.1. Insurance Policy-Ownership Classification

The COIL 2000 challenge established caravan-insurance ownership identification as a cost-sensitive classification problem involving demographic and product-ownership attributes [1,2]. Its low positive prevalence makes majority-class accuracy a poor measure of screening value and motivates evaluation with threshold-sensitive and ranking metrics.

The original benchmark analyses also showed that model selection and cost assumptions materially affect conclusions on this dataset [1,2]. Van der Putten and van Someren [1] further reported that the between-partition difference of the official challenge separation dominates model choice on this benchmark, and Elkan [2] showed that apparently large accuracy differences on COIL 2000 are frequently artifacts of the evaluation design. Accordingly, the present study reports multiple complementary metrics, selects thresholds using validation data, and evaluates variability across training seeds and independent stratified splits. Official and grouped partitions that keep duplicate records together are defined in Section 2.3. Reviews of machine learning in actuarial practice [12] and statistical treatments of actuarial learning [13] describe the same tension between predictive flexibility and auditability that governs how such a model may be used.

#### 1.1.2. Deep Learning for Tabular Data

Tabular data—structured tables with heterogeneous column types—present a distinct challenge for deep learning. Unlike images and text, tabular features lack spatial or sequential locality, and the signal-to-noise ratio per column varies widely. Shwartz-Ziv and Armon [5] benchmarked gradient-boosted trees against multi-layer perceptrons across dozens of classification tasks and concluded that ensembles retain a significant edge on most datasets. Gorishniy et al. [6] reached a similar conclusion but noted that Transformer-based architectures narrow the gap when equipped with proper embedding layers. Borisov et al. [14] surveyed the rapidly expanding literature and identified attention, self-supervised pre-training, and hybrid tree–neural architectures as the three most promising directions.

Two architectures deserve particular mention. TabNet [15] applies sequential attention to select features at each decision step, achieving interpretability comparable to gradient-boosted trees while matching or exceeding their accuracy on several benchmarks. SAINT [16] combines row-wise and column-wise self-attention with contrastive pre-training, showing that inter-sample attention can capture distributional patterns missed by per-sample models. Huang et al. [17] proposed TabTransformer, which contextualizes categorical embeddings through Transformer layers while leaving continuous features untouched. These studies collectively demonstrate that attention-based architectures can be competitive on tabular data when the embedding and regularization strategies are carefully designed.

#### 1.1.3. Autoencoders and Attention Mechanisms

The autoencoder family has evolved considerably since Hinton and Salakhutdinov’s [7] seminal demonstration that greedy layer-wise pre-training enables deep encoder–decoder networks to learn compact nonlinear embeddings. Vincent et al. [8] introduced denoising autoencoders (DAEs), which corrupt the input and train the network to recover the clean version, promoting robustness. Rifai et al. [9] penalized the Frobenius norm of the encoder Jacobian to obtain contractive autoencoders whose representations are locally invariant to input perturbations. Kingma and Welling [10] reformulated the autoencoder within a variational inference framework, yielding the variational autoencoder (VAE), which enables principled generation and density estimation. In an anomaly detection context, Sakurada and Yairi [18] showed that autoencoder reconstruction error outperforms principal component analysis for detecting sensor faults, while An and Cho [19] used VAE reconstruction probability to rank anomalies, achieving state-of-the-art results on several benchmarks. Zong et al. [20] coupled the bottleneck with a density estimator, and the surveys of Ruff et al. [21] and Pang et al. [22] document how reconstruction-based scores are combined with density or one-class objectives. These methods use reconstruction as the detection signal itself, whereas the present model retains full supervision and uses reconstruction only as an auxiliary training term.

Attention mechanisms, introduced to sequence-to-sequence translation by Bahdanau et al. [23] and generalized to the Transformer architecture by Vaswani et al. [24], have since permeated nearly every deep learning sub-field. In the channel attention domain, Hu et al. [11] proposed the squeeze-and-excitation (SE) block, which pools spatial information into a channel descriptor, passes it through a two-layer bottleneck, and produces a sigmoid gating vector that re-scales feature maps. Woo et al. [25] combined channel and spatial attention in CBAM. Lin et al. [26] introduced the global average pooling concept that underlies many attention designs. Adapting these ideas to tabular autoencoders, where the “channel” dimension corresponds to hidden-layer units rather than convolutional feature maps, is conceptually straightforward but requires careful handling of the reduction ratio and the interplay between gating and the reconstruction loss. The present model embeds a channel attention gate in the encoder of a symmetric autoencoder and jointly optimizes classification and reconstruction.

The present framework is related to jointly supervised and reconstructive representation learning. Le et al. [27] showed, in the linear case, how a reconstruction term can regularize a supervised objective; no equivalent guarantee is assumed here for the nonlinear residual architecture. Ghosh et al. [28] regularized deterministic latent codes, while Mondal et al. [29] performed minority oversampling in a class-preserving latent space. Tian et al. [30] combined channel attention with entropy minimization for unsupervised image novelty detection. In contrast, the present model evaluates a variance-increasing marginal regularizer together with supervised classification, symmetric reconstruction, and fully connected channel attention on imbalanced tabular data. Supervised and regularized deterministic autoencoders are included as direct baselines.

A related line of work regularizes the second-order statistics of a learned representation directly. Barlow Twins [31] penalizes the off-diagonal entries of the embedding cross-correlation matrix, and VICReg [32] pairs a hinge on the per-dimension standard deviation with an explicit covariance term, so that variance is maintained and dimensions are decorrelated by separate penalties. Equation (6) below corresponds to the variance component of that design only: it acts on marginal variances and leaves the off-diagonal covariance unconstrained. Section 3.2 therefore reports the covariance spectrum as a diagnostic instead of assuming that increasing marginal variance decorrelates the code. Covariance-aware penalties remain a natural comparison for later work. Broader accounts of what such objectives control are given in the representation-learning review of Bengio et al. [33].

#### 1.1.4. Summary of Research Gaps

Prior studies examine supervised reconstruction, latent regularization, and attention under different objectives and data modalities. Their joint use for imbalanced insurance policy-ownership classification has received limited empirical study. We therefore evaluate whether combining an auxiliary symmetric reconstruction branch, hidden-unit channel attention, and a marginal log-variance penalty improves performance under a common tuning protocol. The contribution lies in the integrated architecture and its controlled empirical evaluation rather than in a claim that any individual component is new.

## 2. Materials and Methods

This section presents the Attention-based Symmetric AutoEncoder (ASAE) with marginal-variance-regularized feature reconstruction, together with the data, experimental configuration, and baseline models used to evaluate it. Section 2.4, Section 2.5, Section 2.6 and Section 2.7 describe the backbone, attention gate, and loss terms; Section 2.8 and Section 2.9 specify the evaluation and baseline-tuning protocol.

### 2.1. Problem Formulation

Let $D\;=\;{\left\{\left(x_{i},y_{i}\right)\right\}}_{i=1}^{N}$ denote a labeled insurance dataset, where $x_{i}\in R^{d}$ is the feature vector of customer record *i* and $y_{i}\in \left\{0,1\right\}$ is the policy-ownership label. In COIL 2000, $y_{i}=1$ denotes caravan-insurance ownership and $y_{i}\;=\;0$ denotes its absence. Predictors and labels are observed in the same cross-section, so *f* distinguishes current ownership rather than forecasting a subsequent acquisition; no temporal ordering between predictors and labels is available in this benchmark. The goal is to learn a mapping $f:R^{d}\to \left[0,1\right]$ that estimates the positive-class score. The target column is excluded from the 86-column record, leaving d = 85 predictors; the positive prevalence is approximately 6%.

### 2.2. Data Description and Preprocessing

The COIL 2000 dataset was originally assembled for the 2000 Computational Intelligence and Learning prediction challenge [1,2]. It contains 9822 customer records described by 86 attributes in total. Following the official University of California, Irvine (UCI) data dictionary, attributes 1–43 are sociodemographic variables derived from zip-code area statistics (e.g., customer subtype, average age, religious composition, and purchasing-power class, the last of which is attribute 43); attributes 44–64 record the monetary contribution level to each of 21 insurance product categories; attributes 65–85 record the corresponding number of policies held for the same 21 product categories; and attribute 86 (CARAVAN, number of mobile home policies) is the binary target, excluded from the input vector, yielding 43+21+21 = 85 predictor attributes. The official UCI distribution ships the data as a 5822-record training file with the target included (ticdata2000.txt) and a 4000-record evaluation file whose predictors and targets are stored in separate files (ticeval2000.txt and tictgts2000.txt); we join the evaluation predictors with their target file on row order and concatenate the two parts to obtain the full 9822-record dataset before performing our own stratified split. All features are integer-coded; sociodemographic attributes use ordinal or nominal encodings, while product and contribution columns are counts or discretized monetary bins. Two attributes are nominal according to the UCI dictionary: the customer subtype (MOSTYPE, attribute 1) and the customer main type (MOSHOOFD, attribute 5); the remaining 83 attributes are ordinal bins, counts, or discretized monetary levels. In the primary shared representation used for the main comparison, all integer codes—including the two nominal attributes—are standardized, reconstructed under an ordinary Euclidean mean-squared-error loss, and interpolated by SMOTE as if they were continuous quantities, which can generate fractional, non-existent category values. Because these choices affect both the training data and the reconstruction objective, the complete pipeline is additionally rerun under a type-consistent representation (one-hot encodings for the two nominal attributes, SMOTENC oversampling, and cross-entropy reconstruction for the discrete blocks), reported in Section 3.5.

Preprocessing for the primary representation proceeds in two stages. All 85 predictor attributes are retained in their original integer-coded form—no additional one-hot expansion is applied to the nominal variables—so the model input dimension is d = 85, with the 86th attribute (CARAVAN) used exclusively as the target label $y_{i}$. All features are then standardized to zero mean and unit variance using training-set statistics to prevent data leakage. In the primary protocol, the dataset is split into training (70%), validation (15%), and test (15%) subsets through stratified sampling, preserving the original class ratio in each partition. Table 1 summarizes the resulting partition sizes; Section 2.3 defines three additional partitioning protocols that isolate exact-duplicate and sociodemographic overlap.

### 2.3. Partitioning Protocols

The 9822 records are not all distinct. Only 8261 distinct 85-dimensional predictor vectors occur, so 2665 records (27.1%) share their entire predictor vector with at least one other record, and the sociodemographic block (attributes 1–43) is coarser still because it is derived from zip-code area statistics rather than from individual customers. Under the primary stratified split, this structure produces overlap between partitions: 311 of the 1473 test records (21.1%) have an exact duplicate in the training partition, and 1308 test records (88.8%) share their 43-dimensional sociodemographic profile with at least one training record. A stratified random split therefore measures performance under partial repetition of training inputs, which is a meaningful operating condition for scoring a customer base drawn from the same zip-code areas but is not equivalent to generalization to unseen customer profiles.

To distinguish these regimes, we define four partitioning protocols.

- Primary (default): The stratified 70/15/15 split of Table 1, used for the main comparison, the ablations, and the imbalance analysis, and regenerated five times with independent split seeds in Section 3.4.
- Official: The separation defined by the COIL 2000 challenge, in which the 5822-record training file forms the development data and the official 4000-record evaluation set (238 positives) is held out as the test set. A stratified 20% of the development records is reserved for validation and threshold selection.
- Group-exact: A grouped split in which the 8261 distinct predictor vectors are the sampling units, so every set of exactly duplicated records is assigned to a single partition.
- Group-demographic: A grouped split on the 43-dimensional sociodemographic profile, which additionally prevents customers from the same zip-code profile from appearing on both sides of the partition.

Positive prevalence stays within 0.1 percentage points of the 5.97% base rate in every partition of every protocol, and the partition index sets are disjoint. In the grouped protocols, no group identifier appears on both sides of a split. The official and grouped partitions isolate the overlap described above. All quantitative results use the primary protocol.

### 2.4. Network Architecture

**Figure 1:**
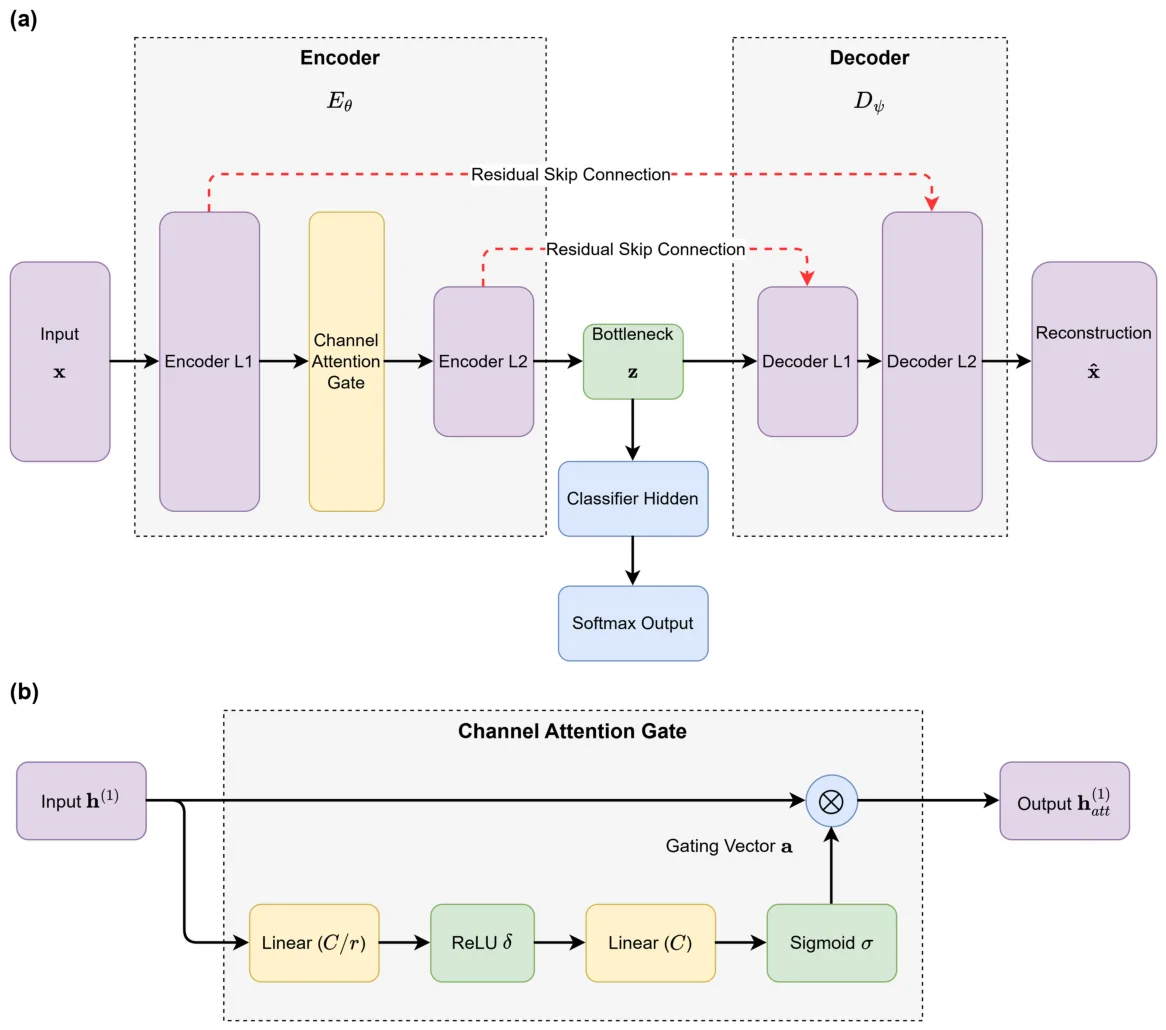
Overall network structure of the proposed Attention-based Symmetric AutoEncoder (ASAE). (**a**) Architecture of the ASAE. Solid arrows denote the forward pass; dashed arrows indicate residual skip connections between symmetric encoder and decoder layers. (**b**) Internal structure of the channel attention gate: the hidden input vector is squeezed through a bottleneck of reduction ratio r=8, producing a sigmoid gating vector that element-wise scales the original activation.

The ASAE consists of four functional blocks: an encoder $E_{\theta }$, a channel attention gate $A_{\phi }$, a decoder $D_{\psi }$ that mirrors the encoder layer dimensions, and a classification head $C_{\omega }$ attached to the bottleneck. Figure 1a illustrates the overall data flow, and Figure 1b shows the internal structure of the channel attention gate.

The encoder maps the 85-dimensional input through two fully connected layers with batch normalization [34] and ReLU activation, reducing dimensions to 128 and then to 64. After the first layer, the channel attention gate (described in Section 2.5) reweights the 128-dimensional hidden vector. Dropout with a probability of p = 0.3 is applied after each encoder layer to prevent co-adaptation of hidden units [35]. A final linear projection compresses the 64-dimensional vector to the 32-dimensional bottleneck z.

The decoder mirrors the encoder: it expands z back through layers of dimension 64 and 128 before producing the reconstruction $\hat{x}\in R^{85}$. Residual connections [36] link each encoder layer to its symmetric decoder counterpart, supplying the decoder with skip information that eases the reconstruction task and provides additional gradient pathways during backpropagation. Because these skip connections allow reconstruction information to bypass the 32-dimensional bottleneck entirely, a low reconstruction loss on its own is not sufficient evidence that class-relevant information is retained inside z; we return to this caveat when interpreting the ablation study in Section 3. The classification head takes z as input, passes it through a 16-unit hidden layer with ReLU, and outputs a two-class softmax probability.

The choice of a relatively compact bottleneck is deliberate. In a small and imbalanced tabular dataset, a wide latent representation can memorize majority-class regularities without improving minority-class recognition. The 32-dimensional code keeps the representation expressive enough to retain cross-feature interactions, while still imposing a compression pressure that makes the reconstruction objective meaningful. The attention gate is placed after the first encoder layer rather than at the bottleneck because early hidden units still preserve a close relationship with groups of input attributes. Reweighting them at this stage therefore affects both the downstream classifier and the reconstruction pathway, whereas a gate applied only to z would mainly act on already-compressed features.

During inference, only the encoder, attention gate, and classification head are needed to produce the ownership score. The decoder serves as an auxiliary branch during training and is not required to score new records.

### 2.5. Channel Attention Module

The channel attention module operates on the 128-dimensional output $h^{\left(1\right)}$ of the first encoder layer. Its purpose is to produce a gating vector $a\in {\left(0,1\right)}^{128}$ that scales each hidden unit according to its relevance to the classification objective. The computation follows the squeeze-and-excitation paradigm [11] adapted to a fully connected setting. Figure 1b (reproduced from Section 2.4) details the module.

Formally, let $h^{\left(1\right)}\;=\;\mathrm{ReLU}\left(\mathrm{BN}\left(W_{\mathrm{enc}}^{\left(1\right)}x+b_{\mathrm{enc}}^{\left(1\right)}\right)\right)$. The attention gate computes

(1) $$a\;=\;\sigma \;\left(W_{2}\;\delta \;\left(W_{1}h^{\left(1\right)}+b_{1}\right)+b_{2}\right),$$

where $W_{1}\in R^{(m/r)\times m}$, $W_{2}\in R^{m\times (m/r)}$, m = 128, reduction ratio r = 8, δ(·) denotes ReLU, and σ(·) denotes the element-wise sigmoid function. The attended hidden vector is then

(2) $$h_{\mathrm{att}}^{\left(1\right)}\;=\;a⊙h^{\left(1\right)},$$

where ⊙ is the Hadamard product. The gate is learned jointly with the classifier and reweights hidden dimensions in a sample-dependent manner. These weights describe internal gating behaviour and are not interpreted as direct importance scores for individual input features.

### 2.6. Symmetric Feature Reconstruction

The decoder $D_{\psi }$ reconstructs $\hat{x}$ from the bottleneck z. Let $h_{\mathrm{dec}}^{\left(k\right)}$ denote the *k*-th decoder hidden layer and $h_{\mathrm{enc}}^{\left(k\right)}$ its symmetric encoder counterpart. The residual connections add the encoder activation to the corresponding decoder layer:

(3) $${\tilde{h}}_{\mathrm{dec}}^{\left(k\right)}\;=\;h_{\mathrm{dec}}^{\left(k\right)}+h_{\mathrm{enc}}^{\left(K-k+1\right)},\;k\;=\;1,\ldots ,K,$$

where *K* is the number of encoder layers. The reconstruction loss is the mean squared error between input and output:

(4) $$L_{\mathrm{recon}}\;=\;\frac{1}{N}\sum_{i\;=\;1}^{N}{∥x_{i}-{\hat{x}}_{i}∥}_{2}^{2}.$$

Equation (4) sums the squared error over the *d* feature dimensions and averages over the *N* records of the mini-batch; it is not averaged over feature dimensions. The implementation uses this reduction, so the weight α in Equation (7) acts on a per-record squared error whose scale is directly comparable with the per-record classification loss of Equation (5).

The reconstruction term is an auxiliary training objective applied jointly with classification loss. Because the residual connections allow information to bypass the bottleneck, reconstruction error is not used as evidence that the complete input distribution is encoded in z. Its contribution is instead evaluated through the decoder-removal and skip-connection ablations reported in Section 3.2. In the type-consistent rerun of Section 3.5, the Euclidean term in Equation (4) applies to the standardized numeric block only, while each one-hot nominal block is reconstructed under a softmax cross-entropy loss, so category codes are never compared by Euclidean distance.

### 2.7. Loss Function and Training Procedure

The classification head outputs a probability vector ${\hat{y}}_{i}\;=\;\left[{\hat{y}}_{i,0},{\hat{y}}_{i,1}\right]$ via softmax. The classification loss is the weighted binary cross-entropy:

(5) $$L_{\mathrm{cls}}\;=\;-\frac{1}{N}\sum_{i\;=\;1}^{N}\left[w_{1}\;y_{i}\log{\hat{y}}_{i,1}+w_{0}\;\left(1-y_{i}\right)\log{\hat{y}}_{i,0}\right],$$

where $w_{1}$ and $w_{0}$ are inverse-frequency class weights that up-weight the minority positive class.

In addition to reconstruction loss, the model uses a marginal log-variance regularizer on the latent bottleneck. It penalizes dimensions with very low mini-batch variance. Under a univariate Gaussian approximation, marginal differential entropy is proportional to log variance, motivating the following tractable objective:

(6) $$L_{\mathrm{ent}}\;=\;-\frac{1}{d_{z}}\sum_{k\;=\;1}^{d_{z}}\log\;\left({\mathrm{Var}}_{B}\left(z_{k}\right)+\varepsilon \right),$$

where $d_{z}\;=\;32$, ${\mathrm{Var}}_{B}\left(z_{k}\right)$ is the empirical mini-batch variance of latent dimension *k*, and $\varepsilon \;=\;10^{-8}$. Minimizing $L_{\mathrm{ent}}$ increases the average log variance of individual dimensions. It does not estimate the joint differential entropy of the latent vector.

Equation (6) uses only diagonal variances and therefore neither captures latent covariance nor guarantees high effective rank. It is also scale-sensitive and class-agnostic, and training-time dropout contributes stochastic variation that is absent at inference. We consequently interpret it only as a marginal log-variance regularizer. Section 3.2 reports effective-rank, covariance-spectrum, class-conditional variance, and latent-norm diagnostics to characterize its observed behaviour.

The total training objective combines all three terms:

(7) $$L\;=\;L_{\mathrm{cls}}+\alpha \;L_{\mathrm{recon}}+\beta \;L_{\mathrm{ent}}+\lambda {∥\Theta ∥}_{2}^{2}.$$

Here, α weights reconstruction, β weights marginal log-variance regularization, λ is the $L_{2}$ coefficient, and Θ = {θ,ϕ,ψ,ω} denotes the trainable parameters. The model is optimized with AdamW [37], the decoupled-weight-decay variant of Adam [38], so the ${\lambda ∥\Theta ∥}_{2}^{2}$ term of Equation (7) is realized as decoupled weight decay in the update rule rather than as an explicit additive loss term; $L_{\mathrm{cls}}$ and $L_{\mathrm{recon}}$ are the only quantities differentiated through the network. Training uses an initial learning rate of $10^{-3}$ with cosine annealing to $10^{-6}$ over a fixed budget of 200 epochs, and the reported checkpoint is the epoch with the highest validation AUC over that budget; early stopping is available with a patience of 20 epochs but is disabled in the reported experiments, so every run completes the full budget. Weights follow Glorot initialization [39]; Table 2 lists the complete configuration.

Validation AUC is used for checkpoint selection because accuracy is dominated by the majority class. The decision threshold is selected on the validation set to maximize the F1-score and then fixed for test evaluation. All neural models use the same split, class-weighting rule, optimizer family, and checkpoint-selection criterion, reducing confounding from different training protocols.

### 2.8. Experimental Setup

All experiments run on a workstation equipped with an NVIDIA RTX 3090 graphics processing unit (GPU, 24 GB memory; NVIDIA Corporation, Santa Clara, CA, USA), 64 GB of random-access memory (RAM), and an Intel i9-12900K central processing unit (CPU; Intel Corporation, Santa Clara, CA, USA). The ASAE and all neural baselines are implemented in PyTorch 2.1 [40]; classical machine learning baselines use scikit-learn 1.3 [41], and oversampling uses imbalanced-learn 0.11. Each experiment is repeated five times with random seeds {0,1,2,3,4}; within a given run, the ASAE and every baseline share the same seed, so the five runs form matched pairs across models. We report the mean and standard deviation (SD) of each metric across the five runs. Statistical comparisons between the ASAE and each baseline use exact paired *t*-tests computed from the five per-seed paired differences (rather than reconstructed from the two marginal SDs), together with 95% confidence intervals for the mean differences based on the *t* distribution with four degrees of freedom. Because twelve baselines are compared on two primary metrics (F1-score and AUC), the family-wise error rate is controlled with the Holm–Bonferroni procedure over the resulting 24 tests, and Holm-adjusted *p*-values are reported. Per-seed predictions, split indices, and the associated summary metrics are provided in the repository listed in the Data Availability Statement.

Inference is deterministic: records that share an identical 85-dimensional predictor vector receive the same score within a given run. The values in the results tables of Section 3 are computed from the per-seed predictions.

To address the severe class imbalance in the training partition (positive rate: 5.98%), we apply the synthetic minority oversampling technique (SMOTE) [42] to all models during training. SMOTE synthesizes new minority-class instances by linear interpolation among existing minority-class nearest neighbors until the minority-to-majority ratio in the training set reaches 1:3. SMOTE is fitted on the training partition only, after standardization with training-set statistics; the validation and test partitions remain unmodified throughout to ensure unbiased evaluation. The primary protocol therefore holds the stratified split fixed and varies the training seed; Section 3.4 complements it with an analysis over five independent stratified re-splits and a factorial comparison of imbalance-handling configurations (with and without SMOTE and class weighting), and Section 3.5 repeats the complete pipeline under a type-consistent representation in which SMOTE is replaced by SMOTENC.

### 2.9. Baseline Methods

We compare the ASAE against twelve baselines spanning three categories. Classical models include Logistic Regression (LR), a Support Vector Machine with an RBF kernel (SVM) [43], Random Forest with 200 trees (RF) [44], XGBoost [3], and LightGBM [4]. The neural baseline is a four-layer multi-layer perceptron (MLP) with the same hidden dimensions as the ASAE encoder (128–64–32). Representation-learning baselines include a standard symmetric autoencoder followed by MLP classification (AE + MLP), a denoising autoencoder with Gaussian noise injection (DAE + MLP) [8], a variational autoencoder (VAE + MLP) [10], a supervised autoencoder trained jointly on cross-entropy and reconstruction losses (SAE) [27], a regularized deterministic autoencoder with an $L_{2}$ penalty on the latent code followed by MLP classification (RAE + MLP) [28], and TabNet [15]. SAE and RAE + MLP share the ASAE encoder–decoder dimensions but contain neither the channel attention gate nor the entropic regularizer, so they represent the closest previously published supervised and regularized autoencoder families identified in Section 1.1.

All models receive the same tuning budget: 50 iterations of Bayesian optimization using a tree-structured Parzen estimator (Optuna 3.5) to maximize the validation AUC. The test set is not used for model selection. Table 3 lists the tuned parameters and search ranges. Neural models share the optimizer family, learning-rate schedule, batch size, checkpoint-selection criterion, class weighting, and SMOTE configuration. This protocol reduces training-procedure confounding while preserving model-specific architectural differences. Unlisted parameters retain the stated library defaults (scikit-learn 1.3, XGBoost 2.0, LightGBM 4.1, pytorch-tabnet 4.1). The comparison covers the classical, neural, and autoencoder families that are directly relevant to the proposed architecture. SAINT [16] and TabTransformer [17], discussed in Section 1.1.2 as context for attention on tabular data, are not included: both rely on large categorical vocabularies and on contrastive or masked pre-training, which do not match the 85 integer-coded columns and 6876 training records used here.

## 3. Results

The experiments are organized around the three contributions stated in Section 1. The main baseline comparison (Section 3.1), the robustness analyses over data splits and imbalance-handling configurations (Section 3.4), and the type-consistent preprocessing rerun (Section 3.5) address Contribution (3); the component ablation, latent-space diagnostics, latent-dimension sensitivity, and t-distributed stochastic neighbor embedding (t-SNE) visualization (Section 3.2, Section 3.3, and Section 3.6.2) address Contribution (1); and the attention ablation row together with the attention-weight analysis (Section 3.6.3) addresses Contribution (2).

### 3.1. Main Results

Table 4 reports test-set performance across five evaluation metrics. On the imbalanced COIL 2000 data, accuracy alone is misleading—a trivial majority-class predictor would score 0.94—so we focus on F1-score and the AUC as the primary metrics.

All paired statistics are computed from the five per-seed differences. Against TabNet, the mean F1-score difference is 0.0639 (difference SD 0.0169), giving t(4) = 8.45, two-sided $p=1.1\times 10^{-3}$, and a 95% confidence interval of [0.043,0.085]. The mean AUC difference is 0.0325 (difference SD 0.0122), giving t(4)=5.96, $p\;=\;4.0\times 10^{-3}$, and a 95% confidence interval of [0.017,0.048]. TabNet yields the smallest contrast of the twelve baselines on both metrics; after Holm–Bonferroni correction over the 24 baseline–metric comparisons, every adjusted *p*-value remains below 0.01.

Among classical models, XGBoost reaches the highest F1-score (0.5250) and AUC (0.8158), consistent with the well-documented strength of gradient-boosted trees on structured data [5]. The gap between XGBoost and logistic regression (AUC difference of 0.097) underscores the importance of modeling nonlinear feature interactions in insurance data. Among autoencoder variants, VAE + MLP and DAE + MLP outperform AE + MLP, suggesting that latent-space regularization—whether through Kullback–Leibler (KL) divergence or input corruption—is associated with improved downstream classification on this benchmark. The supervised autoencoder (SAE) and the regularized deterministic autoencoder (RAE + MLP), which represent the closest previously published joint-objective and latent-regularization families, reach AUC values of 0.8120 and 0.8066, respectively—above the unsupervised autoencoder pipelines but below both TabNet and the ASAE, indicating that joint supervision or generic latent regularization alone does not account for the full ASAE margin. TabNet’s sequential attention mechanism pushes performance further (AUC 0.8259), yet it still falls short of the proposed ASAE (AUC 0.8584).

The ASAE’s advantage is most visible in recall and precision simultaneously. While some baselines achieve reasonable precision by being highly selective (e.g., XGBoost at 0.60), they sacrifice recall; the ASAE maintains precision at 0.62 while lifting recall to 0.5793, resulting in an F1-score that exceeds TabNet by 0.064 points. A model that separates policyholders from non-policyholders at both ends of this trade-off is useful for portfolio screening and for prioritizing follow-up contact, but the balance that is preferable in practice depends on the cost attached to each error type.

The absolute differences should also be interpreted in light of the severe class imbalance. In the representative runs corresponding to the displayed operating points, the ASAE identifies 51 of 87 positive cases, compared with 43 for TabNet, while maintaining higher precision. These counts illustrate the reported precision–recall difference but do not isolate the effect of any single architectural component.

Accuracy changes are more modest because the negative class dominates the sample. For instance, several baselines already exceed 0.94 accuracy while still missing most positive cases. The stronger separation appears in F1-score and the AUC, which are less affected by the majority-class prior. For this reason, the ASAE’s 0.0325 AUC gain over TabNet and 0.0426 AUC gain over XGBoost are more informative than the smaller differences in accuracy.

**Figure 2:**
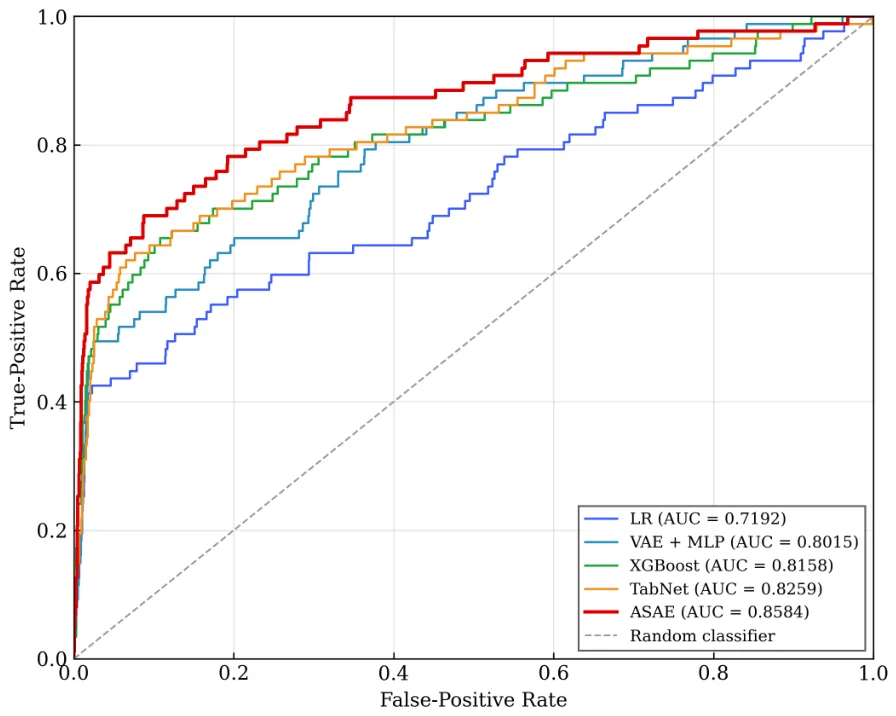
ROC curves for five representative models on the COIL 2000 test set. The dashed diagonal line denotes a random classifier (AUC = 0.5). The AUC values correspond to the five-run means reported in Table 4.

Figure 2 displays the receiver operating characteristic (ROC) curves for five representative models.

The ASAE has the highest AUC among the representative curves. Its curve is also above the displayed baselines across much of the low-to-moderate false-positive range. This region is the relevant one when only a limited number of records can be reviewed, although operational usefulness requires an application-specific cost analysis.

### 3.2. Ablation Study

We train six ablated variants: without the marginal log-variance regularizer; without channel attention; without the reconstruction branch while retaining $L_{\mathrm{ent}}$; without residual connections; without dropout; and an encoder-only classifier with neither decoder nor $L_{\mathrm{ent}}$. The reconstruction-free variant removes the decoder and all skip connections, whereas the no-residual variant reconstructs from z alone. Panel A of Table 5 reports the ablations, and Panel B of Table 5 reports latent-dimension sensitivity.

Removing $L_{\mathrm{ent}}$ reduces the AUC from 0.8584 to 0.8456 and the F1-score from 0.6008 to 0.5799. The covariance diagnostics below examine whether this performance difference coincides with a less concentrated latent representation.

Removing the reconstruction branch reduces the AUC from 0.8584 to 0.8253 and the F1-score from 0.6008 to 0.5386, the largest change on both metrics among the three principal component ablations; removing channel attention is next (AUC −0.030, F1-score −0.056), and removing $L_{\mathrm{ent}}$ has the least effect (AUC −0.013, F1-score −0.021). Removing dropout is not a component of the proposed architecture but produces the lowest values in the panel (F1-score 0.5175, AUC 0.8152), which indicates that the configuration depends on regularization strength as well as on architecture. The no-residual variant retains more performance than the reconstruction-free variant, but its skip-free design also changes the information path through the decoder. The encoder-only model reaches an AUC of 0.8188, compared with 0.8253 when $L_{\mathrm{ent}}$ is retained without reconstruction. This ordering is consistent with a marginal contribution from the log-variance term, although no causal mechanism is inferred from the ablation alone.

Across the tested variants, the complete configuration records the highest F1-score and AUC. Accuracy does not follow the same ordering—the variant without $L_{\mathrm{ent}}$ records the highest accuracy in the panel (0.9561)—because accuracy is dominated by the 94% negative class and moves with the selected threshold rather than with ranking quality. The ablations therefore support retaining all three components under this evaluation protocol, without establishing that they act through independent causal mechanisms.

The latent diagnostics use the participation ratio $\mathrm{PR}={\left(\sum_{k}\lambda_{k}\right)}^{2}/\sum_{k}\lambda_{k}^{2}$ of the eigenvalues $\lambda_{k}$ of the test-set bottleneck covariance, reported as the mean ± SD over the five runs. The full ASAE reaches a PR = 22.9±0.6 out of 32 dimensions, with its top five eigenvalues accounting for 35.8%±0.7% of total variance. Without $L_{\mathrm{ent}}$, the values are 16.1±0.3 and 48.1%±0.6%, and the encoder-only variant reaches 14.0±0.4 and 52.7%±0.9%. Positive-class mean per-dimension variance is approximately 41% higher under the full objective than without $L_{\mathrm{ent}}$ (0.363 against 0.257). The mean bottleneck norm stays within ±6% of its epoch-50 value, showing no unbounded scale growth during the observed training window. These are descriptive associations and do not remove the scale sensitivity of Equation (6).

### 3.3. Sensitivity to Latent Dimension

The bottleneck dimensionality controls the information–compression trade-off. The latent dimension of 32 used throughout this paper was selected on validation AUC before any test-set evaluation; Panel B of Table 5 reports test-set performance across candidate dimensions from 8 to 64 as a post hoc sensitivity analysis only, not as a basis for re-selecting the operating dimension.

Among the tested dimensions, 32 gives the highest validation-selected test performance. Performance is lower at both smaller and larger dimensions; the experiment does not isolate whether compression, optimization, or overfitting explains these differences.

The lower results at 48 and 64 dimensions are moderate but consistent across the reported means. The 32-dimensional setting is therefore retained as the validation-selected operating point for this benchmark.

### 3.4. Robustness to Data Partitioning and Imbalance Handling

The primary protocol of Section 2.8 holds the stratified split fixed and varies only the training seed, so the SDs in Table 4 capture optimization randomness but not sampling variability. Because the test partition contains only 87 positive cases, recall-dependent metrics may be sensitive to which positives fall into the test set. We therefore regenerated five independent stratified 70/15/15 splits (split seeds 10–14, one training run per split with a fixed training seed) and repeated the comparison for the three strongest models; all preprocessing, including standardization and SMOTE, was refitted within the training partition of each split. Table 6 reports the results.

Across-split SDs are two to four times the seed-only SDs in Table 4, indicating greater variability across these five partitions than across the five training seeds. The ASAE–TabNet F1-score difference remains positive on every tested split, with a minimum gap of 0.048. The ASAE–XGBoost difference is positive on four splits and marginally negative on one (−0.005), and the XGBoost across-split SD of 0.0569 is the largest in the table, so the two models are not separated on F1-score by this analysis; the AUC ordering is unchanged on all five splits.

Second, because SMOTE and class-weighted cross-entropy both target class imbalance, we disentangle their contributions from that of the architecture with a 2×2 factorial comparison: (i) neither mechanism, (ii) class weighting only, (iii) SMOTE only, and (iv) both (the default configuration). Table 7 reports the results for the ASAE and TabNet under the primary fixed-split protocol.

For the ASAE, each mechanism raises the AUC on its own and the combination gives the highest F1-score and AUC; the F1-score gain is concentrated in the combined configuration (0.545 with neither mechanism against 0.601 with both), whereas either mechanism alone adds about 0.005. For TabNet, the two mechanisms raise the AUC but leave the F1-score within 0.016 of the unadjusted configuration. The ASAE–TabNet AUC difference is stable across the four configurations (0.033–0.039), while the F1-score difference varies more widely (0.016–0.064) and is largest in the default configuration. The imbalance-handling choice therefore affects the size of the F1-score margin, and the margin should be read together with the configuration that produced it.

### 3.5. Type-Consistent Preprocessing Rerun

The primary representation treats the two nominal attributes as continuous integer codes, so distances, reconstruction errors, and SMOTE interpolations depend on an arbitrary category numbering. To test whether the comparison depends on this simplification, the complete pipeline—preprocessing, oversampling, tuning, and training of all thirteen models—was rerun under a type-consistent representation. The customer subtype and customer main type were one-hot encoded using the categories observed in the training partition (40 and 10 indicator columns, respectively), and the remaining 83 numeric attributes were standardized with training-set statistics, giving a 133-dimensional input. Ordinary SMOTE is replaced by SMOTENC with the two nominal columns declared categorical, so synthetic minority records receive observed category codes by neighborhood vote instead of fractional interpolations (ratio 1:3, as before). In the ASAE and the autoencoder baselines, the decoder reconstructs the numeric block under the mean squared error and each nominal block under a softmax cross-entropy loss, so the reconstruction objective no longer penalizes Euclidean deviations between arbitrary category codes. Every model was re-tuned with the same 50-iteration Bayesian optimization protocol on the new representation and retrained with the same stratified split and the same paired seeds {0,1,2,3,4}. Table 8 reports the results.

The model ranking is unchanged relative to Table 4, and the individual differences are moderate: logistic regression benefits most from the one-hot expansion (AUC +0.019), the tree ensembles change little (+0.002 to +0.004 in AUC), and the neural models gain between 0.008 and 0.016 in AUC. Under the type-consistent representation, the ASAE reaches an F1-score of 0.6241 ± 0.0074 and an AUC of 0.8702 ± 0.0050. The paired ASAE–TabNet contrasts computed from the five per-seed differences are 0.0798 in F1-score (difference SD 0.0140, t(4) = 12.76, two-sided $p\;=\;2.2\times 10^{-4}$, 95% confidence interval [0.062,0.097]) and 0.0357 in AUC (difference SD 0.0050, t(4)=15.93, $p\;=\;9.1\times 10^{-5}$, 95% confidence interval [0.029,0.042]); all 24 baseline–metric comparisons remain significant after Holm–Bonferroni correction (adjusted p<0.002). The observed ASAE margin is therefore not attributable to the integer-coded treatment of the nominal attributes.

### 3.6. Visualization Analysis

#### 3.6.1. Training Dynamics

**Figure 3:**
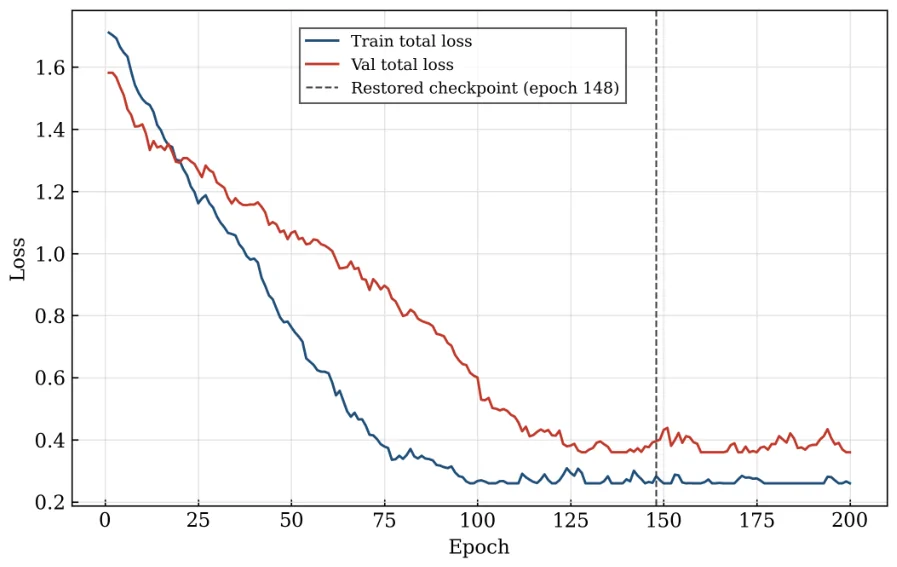
Training and validation total loss curves for the ASAE over the full 200-epoch monitoring window. The dashed line marks epoch 148, from which the best checkpoint was restored based on validation AUC; the loss curves continue beyond this point for diagnostic monitoring only.

Figure 3 shows the training and validation losses recorded over a 200-epoch diagnostic run. Model selection uses the checkpoint with the highest validation AUC; for this run, that checkpoint occurs at epoch 148. Continuing the diagnostic trace does not alter the selected checkpoint or use test information.

#### 3.6.2. Latent Space Visualization

**Figure 4:**
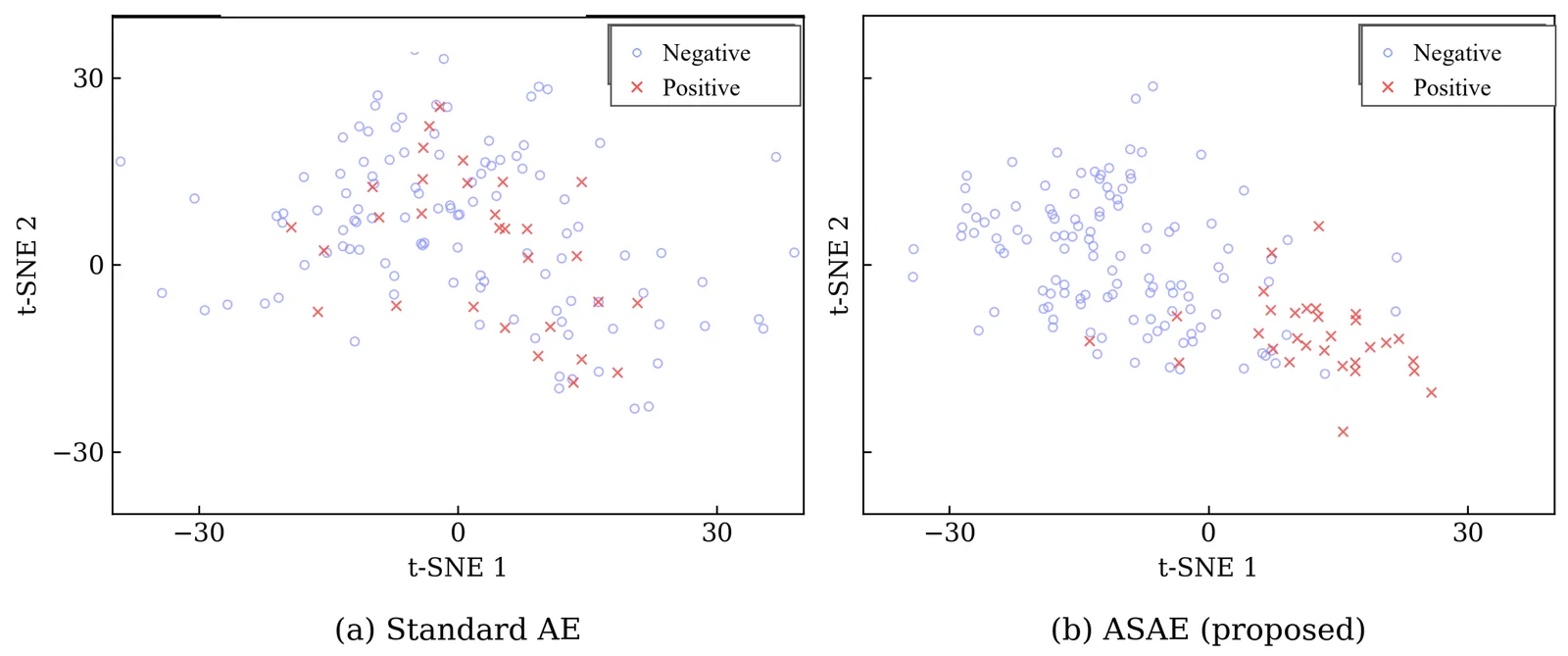
t-SNE visualization of a class-enriched subsample of the test-set representations. The displayed class ratio does not represent the 5.9% test prevalence. (**a**) Standard AE. (**b**) ASAE.

To assess whether the ASAE produces a latent space with meaningful class structure, we apply t-SNE [45] (default perplexity 30, learning rate 200, 1000 iterations) to the 32-dimensional bottleneck representations of the test set. Because the positive class constitutes only 5.9% of the 1473 test records, a class-proportional scatter plot would render the positive cases nearly invisible; Figure 4 therefore plots a deliberately class-enriched subsample of 150 embeddings—all 87 positive test cases together with 63 randomly sampled negative cases—so that both classes are visible. The plotted class ratio is intentional and does not reflect the test-set base rate. Figure 4 compares the latent embeddings of the standard AE and the proposed ASAE.

In the standard AE embedding, positive-class cases are scattered across the negative-class cloud with no visible separation. The ASAE embedding, by contrast, concentrates most positive cases in the lower-right quadrant, forming a recognizable cluster with clearer boundaries. This visual pattern is qualitatively consistent with the quantitative AUC gap between the two models, though t-SNE embeddings are stochastic and non-metric and should be interpreted as illustrative rather than as independent quantitative evidence of separability.

#### 3.6.3. Attention Weight Distribution

**Figure 5:**
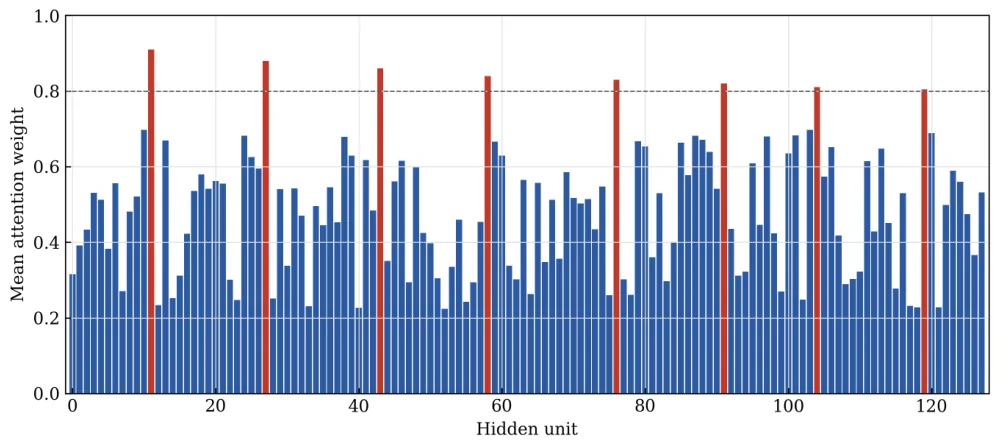
Mean attention weights across the 128 hidden units for positive-class cases. Red bars mark units whose mean weight exceeds the 0.8 reference level (dashed line); blue bars mark the remaining units. Units with high weights (>0.8) encode learned feature combinations that the gate treats as most relevant to the positive class; the weight of a unit does not directly attribute importance to any single raw input feature.

Figure 5 summarizes the sigmoid gate values across the 128 hidden units, averaged over positive test cases. These values describe hidden-unit gating and are not direct feature-importance scores.

Weights range from approximately 0.22 to 0.91, with a small subset above 0.8 and most between 0.3 and 0.7. The non-uniform profile shows that the gate applies different scaling across hidden units. Because each unit combines many input columns, the plot cannot be interpreted as a ranking of raw features. Formal attribution analysis was not performed.

#### 3.6.4. Confusion Matrix

**Figure 6:**
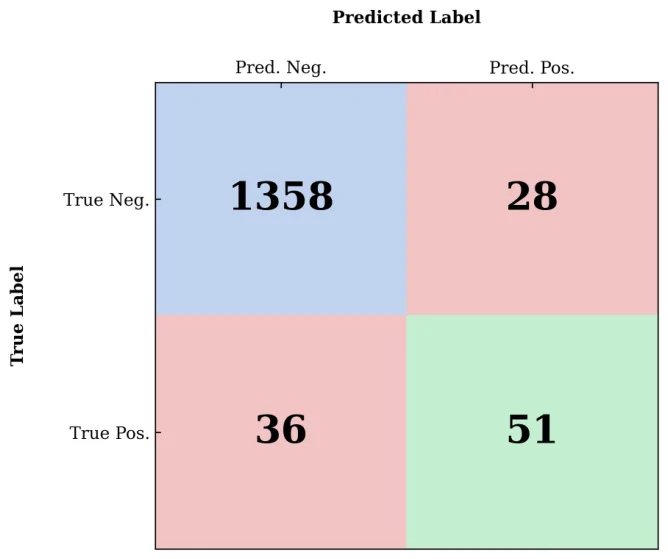
Confusion matrix of the ASAE for a representative run on the COIL 2000 test set (n=1473). Table 4 reports means across five runs.

Figure 6 presents the confusion matrix of the ASAE on the test set.

In the visualized run, the model identifies 51 of 87 positive cases and produces 28 false positives among 1386 negative cases. Accuracy, precision, recall, and F1-score are 0.9566, 0.6456, 0.5862, and 0.6145, respectively. The operational acceptability of this error profile depends on the relative cost of a false positive and a missed policyholder in the intended application.

### 3.7. Comparison of Performance Across Metric Dimensions

**Figure 7:**
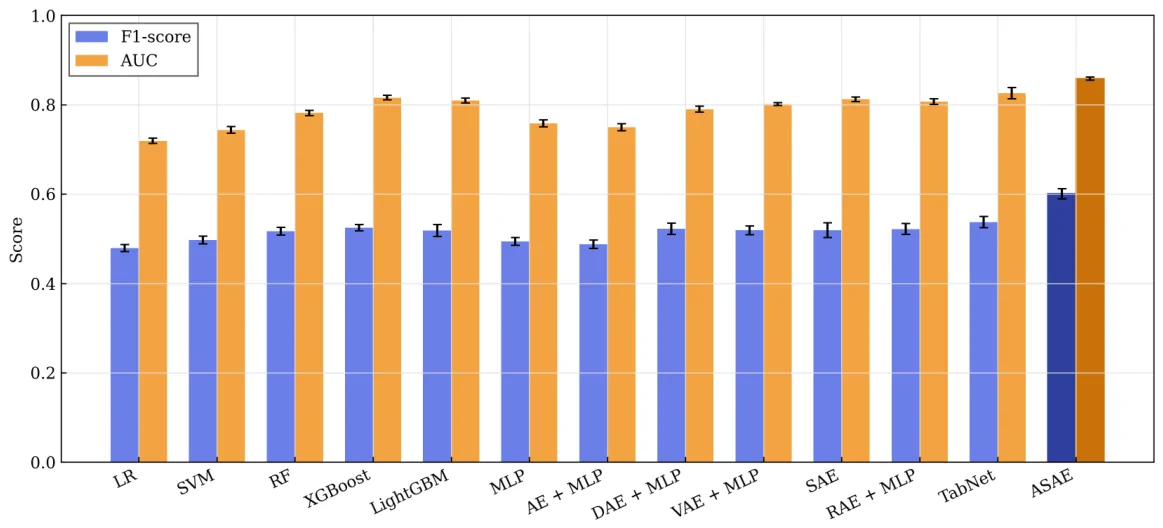
F1-score and AUC comparison across all models, reproduced from the means and SDs in Table 4 (error bars show ±1 SD). The ASAE achieves the highest values for both metrics.

Figure 7 provides a visual summary of the F1-score and AUC means reported in Table 4 for all models.

## 4. Discussion

The results show that the marginal log-variance term is associated with both predictive and covariance-spectrum differences on this benchmark. Removing $L_{\mathrm{ent}}$ reduces the AUC by 0.013, while the participation-ratio effective rank decreases from 22.9 to 16.1 and the top-five eigenvalue share increases. These observations are consistent with a less concentrated latent covariance under the full objective. They do not establish joint-entropy maximization or a general anti-collapse mechanism because the regularizer remains covariance-blind, scale-sensitive, and class-agnostic. Of the three components, this term also carries the smallest predictive difference, so the spectral change is not treated as the main source of the reported margin.

The reconstruction branch is best interpreted as an auxiliary training objective. Removing it reduces the AUC by 0.033 and the F1-score by 0.062, the largest change in both metrics among the three principal component ablations. However, skip connections allow reconstruction information to bypass z, so neither reconstruction error nor this ablation demonstrates that the bottleneck retains the complete input distribution.

Channel attention provides sample-dependent reweighting of hidden units. Removing the gate produces the second-largest F1-score change among the principal component ablations. The non-uniform gate profile confirms that hidden units receive different scaling, but it does not provide raw-feature attribution or substitute for a formal interpretability analysis.

Residual connections add no trainable parameters and are associated with a smaller performance difference than the reconstruction, attention, and dropout ablations. Their precise optimization effect was not isolated in this study.

In applied use, the model should serve as a ranking tool rather than as an automatic decision system, and the operating threshold must be selected from the costs attached to each error type in the intended application. Because SMOTE and class-weighted training can affect probability calibration, the softmax output should not be treated as a calibrated ownership probability without separate calibration analysis.

The attention and latent-space plots are diagnostic summaries of model behaviour. They should not be interpreted as feature-level explanations, fairness evidence, or independent confirmation of class separability.

The study has three principal limitations. COIL 2000 is an older benchmark and may not represent current customer populations or distribution channels. The primary comparison processes nominal integer codes with standardization, Euclidean reconstruction, and ordinary SMOTE; the type-consistent rerun in Section 3.5 shows that the model ranking and the ASAE margin persist under one-hot encodings, SMOTENC oversampling, and categorical reconstruction losses, although learned categorical embeddings and alternative encodings were not evaluated. Finally, the empirical evidence comes from one dataset and one ownership target; external validation is required before deployment.

The re-split analysis indicates that the main comparison is not an artifact of a single partition, but it does not establish general superiority across insurance datasets. All five re-splits are stratified random partitions of the same records, so they quantify sampling variability rather than the effect of the duplicate structure described in Section 2.3. The grouped and official partitions isolate that overlap and correspond to a stricter generalization setting. Across-split variability is two to four times the seed-only variability, underscoring the need for evaluation on additional public and contemporary insurance datasets.

The evaluation does not include precision–recall AUC, calibration, or decision-cost analysis. These analyses are needed before threshold-based operational use. Comparisons with latent-space oversampling [29] and entropy-minimizing attention autoencoders [30] also remain indirect. Code, split indices, and per-run predictions are provided in the repository listed in the Data Availability Statement.

## 5. Conclusions

This paper introduced the Attention-based Symmetric AutoEncoder for imbalanced caravan-insurance policy-ownership classification on the COIL 2000 benchmark. The model combines an auxiliary symmetric reconstruction branch, hidden-unit channel attention, and a marginal log-variance regularizer. The latter is a tractable heuristic applied to individual latent variances, not an estimator of joint differential entropy.

Under the stratified protocol used for the main comparison, the ASAE records an F1-score of 0.6008 ± 0.0115 and an AUC of 0.8584 ± 0.0034, exceeding the twelve baselines considered on this benchmark. The differences relative to TabNet remain positive across the tested stratified re-splits and imbalance-handling configurations, and persist under the complete type-consistent preprocessing rerun (F1-score 0.6241 ± 0.0074, AUC 0.8702 ± 0.0050). Removing the reconstruction branch reduces the AUC by 0.033, removing channel attention reduces the AUC by 0.030, and removing the log-variance term reduces the AUC by 0.013. These ablations describe performance associations on COIL 2000 under the tested protocol.

The covariance diagnostics show that the full model is associated with a higher effective rank and a less concentrated covariance spectrum than the model without $L_{\mathrm{ent}}$. Together with the paired comparisons and robustness analyses, these results support the ASAE configuration on COIL 2000. The reported margins are obtained under stratified random partitioning, in which 21.1% of test records have an exact duplicate in the training partition and 88.8% share a sociodemographic profile with a training record. The conclusions remain conditional on this benchmark, the representation, the tuning protocol, and the baseline implementations.

Future work should evaluate learned categorical embeddings, covariance-aware regularizers, calibration, and decision-cost metrics. Validation on additional insurance datasets, and under partitioning that separates duplicated and demographically identical records, is also necessary to assess generalizability beyond COIL 2000.

## Figures and Tables

**Table 1:** COIL 2000 dataset partition summary.

Partition	Samples	Positive (%)	Negative (%)
Training	6876	411 (5.98%)	6465 (94.02%)
Validation	1473	88 (5.97%)	1385 (94.03%)
Test	1473	87 (5.91%)	1386 (94.09%)
Total	9822	586 (5.97%)	9236 (94.03%)

**Table 2:** Hyperparameter settings for the ASAE model.

Hyperparameter	Value
Encoder layer dimensions	85 → 128 → 64 → 32
Decoder layer dimensions	32 → 64 → 128 → 85
Attention reduction ratio *r*	8
Dropout probability *p*	0.3
Reconstruction weight α	0.5
Marginal log-variance weight β	$10^{-2}$
Weight decay λ	$10^{-4}$ (decoupled)
Learning rate (initial)	$10^{-3}$
Learning-rate schedule	Cosine annealing to $10^{-6}$
Optimizer	AdamW ($\beta_{1}=0.9$, $\beta_{2}=0.999$)
Batch size	256
Training budget (epochs)	200 (fixed; run to completion)
Checkpoint selection	Best validation AUC within the budget
Early stopping patience	20 (available; disabled by default)

**Table 3:** Hyperparameter search spaces used for baseline model selection (50 iterations of Bayesian optimization per model, maximizing the validation AUC; log-uniform priors are marked “log”).

Model	Tuned Hyperparameters (Search Range)
LR	Inverse regularization strength *C* ($10^{-3}$–$10^{2}$, log)
SVM	*C* ($10^{-2}$–$10^{2}$, log); RBF width γ ($10^{-4}$–1, log)
RF	Max. depth (4–32); min. samples per leaf (1–20); max. features (0.2–1.0)
XGBoost/LightGBM	Learning rate ($10^{-3}$–0.3, log); max. depth (3–10); number of trees (100–1000); subsample (0.5–1.0); column subsample (0.5–1.0); min. child weight (1–10)
MLP/AE/DAE/VAE/SAE/RAE	learning rate ($10^{-4}$–$10^{-2}$, log); dropout (0.0–0.5); weight decay ($10^{-6}$–$10^{-3}$, log); DAE noise SD (0.05–0.5); VAE KL weight ($10^{-3}$–1, log); SAE reconstruction weight (0.1–2.0); RAE latent penalty ($10^{-4}$–$10^{-1}$, log)
TabNet	$n_{d}=n_{a}$ (8–64); decision steps (3–10); relaxation γ (1.0–2.0); sparsity λ ($10^{-5}$–$10^{-2}$, log); learning rate ($10^{-3}$–$2\times 10^{-2}$, log)

**Table 4:** Test-set performance comparison under the primary stratified protocol. Best results are in **bold**; second-best are underlined. Accuracy, Precision, and Recall are means over five paired runs; F1-Score and AUC are reported as mean ± SD over five runs.

Model	Accuracy	Precision	Recall	F1-Score	AUC
LR	0.9457	0.5528	0.4230	0.4792 ± 0.0082	0.7192 ± 0.0060
SVM [43]	0.9465	0.5591	0.4483	0.4974 ± 0.0088	0.7435 ± 0.0074
RF [44]	0.9477	0.5691	0.4736	0.5169 ± 0.0090	0.7815 ± 0.0058
XGBoost [3]	0.9499	0.5971	0.4690	0.5250 ± 0.0071	0.8158 ± 0.0049
LightGBM [4]	0.9477	0.5688	0.4759	0.5182 ± 0.0134	0.8094 ± 0.0053
MLP	0.9449	0.5423	0.4552	0.4940 ± 0.0087	0.7583 ± 0.0078
AE + MLP [7]	0.9422	0.5118	0.4667	0.4878 ± 0.0097	0.7499 ± 0.0079
DAE + MLP [8]	0.9466	0.5544	0.4943	0.5224 ± 0.0126	0.7898 ± 0.0068
VAE + MLP [10]	0.9446	0.5329	0.5057	0.5189 ± 0.0101	0.8015 ± 0.0030
SAE [27]	0.9469	0.5582	0.4851	0.5190 ± 0.0164	0.8120 ± 0.0053
RAE + MLP [28]	0.9468	0.5558	0.4920	0.5218 ± 0.0121	0.8066 ± 0.0063
TabNet [15]	0.9473	0.5584	0.5172	0.5369 ± 0.0126	0.8259 ± 0.0123
ASAE (ours)	**0.9545**	**0.6240**	**0.5793**	**0.6008 ± 0.0115**	**0.8584 ± 0.0034**

**Table 5:** Consolidated robustness analysis on the COIL 2000 test set: (Panel A) component ablation and (Panel B) latent-dimension sensitivity. F1-Score and AUC are reported as mean ± SD over five runs. Bold marks the highest F1-score and AUC within each panel; accuracy is reported for completeness and is not used for ranking.

Variant/Latent Dim.	Accuracy	F1-Score	AUC
Panel A: Component Ablation			
ASAE (full model)	0.9545	**0.6008 ± 0.0115**	**0.8584 ± 0.0034**
w/o Marginal-Variance Regularizer	0.9561	0.5799 ± 0.0083	0.8456 ± 0.0055
w/o Channel Attention	0.9537	0.5448 ± 0.0128	0.8283 ± 0.0075
w/o Symmetric Reconstruction	0.9530	0.5386 ± 0.0102	0.8253 ± 0.0074
w/o Residual Connections	0.9553	0.5595 ± 0.0131	0.8364 ± 0.0062
w/o Dropout	0.9517	0.5175 ± 0.0116	0.8152 ± 0.0095
Encoder-only (no decoder)	0.9526	0.5290 ± 0.0142	0.8188 ± 0.0067
Panel B: Latent-Dimension Sensitivity (full ASAE, dimension varied)			
8	0.9508	0.4944 ± 0.0188	0.8061 ± 0.0089
16	0.9553	0.5608 ± 0.0157	0.8377 ± 0.0064
32 (used throughout)	0.9545	**0.6008 ± 0.0115**	**0.8584 ± 0.0034**
48	0.9560	0.5814 ± 0.0178	0.8492 ± 0.0065
64	0.9556	0.5714 ± 0.0118	0.8414 ± 0.0065

**Table 6:** Performance across five independent stratified train/validation/test splits (mean ± SD over splits; one run per split). The rightmost column reports the range of the per-split ASAE–baseline difference.

Model	F1-Score	AUC	Per-Split F1 Gap to ASAE
XGBoost	0.5464 ± 0.0569	0.8119 ± 0.0141	−0.005–0.142
TabNet	0.5199 ± 0.0157	0.8187 ± 0.0153	0.048–0.081
ASAE (ours)	0.5923 ± 0.0234	0.8608 ± 0.0121	—

**Table 7:** Factorial comparison of imbalance-handling configurations (mean ± SD over five paired runs on the fixed split). CW denotes class-weighted cross-entropy. The default configuration is the same as in Table 4.

Configuration	ASAE F1	ASAE AUC	TabNet F1	TabNet AUC
Neither	0.5453 ± 0.0123	0.8450 ± 0.0069	0.5292 ± 0.0167	0.8089 ± 0.0077
CW only	0.5506 ± 0.0142	0.8564 ± 0.0075	0.5216 ± 0.0139	0.8176 ± 0.0062
SMOTE only	0.5509 ± 0.0142	0.8518 ± 0.0061	0.5280 ± 0.0161	0.8174 ± 0.0077
CW + SMOTE (default)	0.6008 ± 0.0115	0.8584 ± 0.0034	0.5369 ± 0.0126	0.8259 ± 0.0123

**Table 8:** Test-set performance under the type-consistent representation (one-hot nominal attributes, SMOTENC oversampling, categorical reconstruction losses). Best results are in **bold**; second-best are underlined. Accuracy, Precision, and Recall are means over five paired runs; F1-Score and AUC are mean ± SD over five runs.

Model	Accuracy	Precision	Recall	F1-Score	AUC
LR	0.9450	0.5437	0.4368	0.4841 ± 0.0108	0.7385 ± 0.0055
SVM	0.9468	0.5617	0.4552	0.5025 ± 0.0070	0.7585 ± 0.0066
RF	0.9476	0.5676	0.4736	0.5162 ± 0.0091	0.7837 ± 0.0066
XGBoost	0.9500	0.6000	0.4690	0.5260 ± 0.0109	0.8197 ± 0.0046
LightGBM	0.9488	0.5814	0.4782	0.5246 ± 0.0067	0.8130 ± 0.0052
MLP	0.9466	0.5649	0.4736	0.5126 ± 0.0133	0.7742 ± 0.0078
AE + MLP	0.9422	0.5128	0.4782	0.4944 ± 0.0114	0.7655 ± 0.0076
DAE + MLP	0.9468	0.5546	0.5034	0.5277 ± 0.0142	0.8004 ± 0.0056
VAE + MLP	0.9454	0.5416	0.5149	0.5273 ± 0.0106	0.8118 ± 0.0064
SAE	0.9472	0.5600	0.4966	0.5262 ± 0.0101	0.8202 ± 0.0053
RAE + MLP	0.9472	0.5594	0.4989	0.5273 ± 0.0151	0.8174 ± 0.0074
TabNet	0.9477	0.5611	0.5287	0.5443 ± 0.0101	0.8345 ± 0.0071
ASAE (ours)	**0.9585**	**0.6728**	**0.5839**	**0.6241 ± 0.0074**	**0.8702 ± 0.0050**

## Data Availability

The COIL 2000 dataset is publicly available from the UCI Machine Learning Repository at https://archive.ics.uci.edu/dataset/125/insurance+company+benchmark+coil+2000 (accessed on 31 August 2026). The train/validation/test split indices, per-seed random states, baseline hyperparameter search spaces and selected hyperparameters, per-seed raw predictions, and training code used to produce Table 4, Table 5, Table 6, Table 7 and Table 8 have been deposited in a public repository at https://github.com/koopman-mpc-lab/asae-coil2000-reproduction (accessed on 31 August 2026), so that the reported table comparisons can be independently recomputed and audited.

## Abbreviations

The following abbreviations are used in this manuscript:

ASAE	Attention-based Symmetric AutoEncoder
AE	Autoencoder
AUC	Area Under the Receiver Operating Characteristic Curve
BN	Batch Normalization
CBAM	Convolutional Block Attention Module

CoIL/COIL	Computational Intelligence and Learning (benchmark dataset)
CPU	Central Processing Unit
DAE	Denoising Autoencoder
GPU	Graphics Processing Unit
IMRaD	Introduction, Methods, Results, and Discussion
KL	Kullback–Leibler (divergence)
LR	Logistic Regression
MLP	Multi-Layer Perceptron
MSE	Mean Squared Error
RAM	Random-Access Memory
RAE	Regularized (Deterministic) Autoencoder
RF	Random Forest
ROC	Receiver Operating Characteristic
SAE	Supervised Autoencoder
SD	Standard Deviation
SE	Squeeze-and-Excitation
SMOTE	Synthetic Minority Oversampling Technique
SMOTENC	SMOTE for Nominal and Continuous Features
SVM	Support Vector Machine
t-SNE	t-distributed Stochastic Neighbor Embedding
UCI	University of California, Irvine
VAE	Variational Autoencoder

## References

[1] Van der Putten P., van Someren M. (2004). A Bias–Variance Analysis of a Real World Learning Problem: The CoIL Challenge 2000. Mach. Learn..

[2] Elkan C. (2001). Magical Thinking in Data Mining: Lessons from CoIL Challenge 2000. Proceedings of the Seventh ACM SIGKDD International Conference on Knowledge Discovery and Data Mining, San Francisco, CA, USA, 26–29 August 2001.

[3] Chen T., Guestrin C. (2016). XGBoost: A Scalable Tree Boosting System. Proceedings of the 22nd ACM SIGKDD International Conference on Knowledge Discovery and Data Mining, San Francisco, CA, USA, 13–17 August 2016.

[4] Ke G., Meng Q., Finley T., Wang T., Chen W., Ma W., Ye Q., Liu T.-Y. (2017). LightGBM: A Highly Efficient Gradient Boosting Decision Tree. Advances in Neural Information Processing Systems 30.

[5] Shwartz-Ziv R., Armon A. (2022). Tabular Data: Deep Learning Is Not All You Need. Inf. Fusion.

[6] Gorishniy Y., Rubachev I., Khrulkov V., Babenko A. (2021). Revisiting Deep Learning Models for Tabular Data. Advances in Neural Information Processing Systems 34.

[7] Hinton G.E., Salakhutdinov R.R. (2006). Reducing the Dimensionality of Data with Neural Networks. Science.

[8] Vincent P., Larochelle H., Lajoie I., Bengio Y., Manzagol P.-A. (2010). Stacked Denoising Autoencoders: Learning Useful Representations in a Deep Network with a Local Denoising Criterion. J. Mach. Learn. Res..

[9] Rifai S., Vincent P., Muller X., Glorot X., Bengio Y. (2011). Contractive Auto-Encoders: Explicit Invariance During Feature Extraction. Proceedings of the 28th International Conference on Machine Learning, Bellevue, WA, USA, 28 June–2 July 2011.

[10] Kingma D.P., Welling M. Auto-Encoding Variational Bayes. Proceedings of the 2nd International Conference on Learning Representations.

[11] Hu J., Shen L., Sun G. (2018). Squeeze-and-Excitation Networks. Proceedings of the IEEE Conference on Computer Vision and Pattern Recognition, Salt Lake City, UT, USA, 18–23 June 2018.

[12] Richman R. (2021). AI in Actuarial Science—A Review of Recent Advances—Part 2. Ann. Actuar. Sci..

[13] Wüthrich M.V., Merz M. (2023). Statistical Foundations of Actuarial Learning and Its Applications.

[14] Borisov V., Leemann T., Seßler K., Haug J., Pawelczyk M., Kasneci G. (2024). Deep Neural Networks and Tabular Data: A Survey. IEEE Trans. Neural Netw. Learn. Syst..

[15] Arık S.Ö., Pfister T. (2021). TabNet: Attentive Interpretable Tabular Learning. Proceedings of the AAAI Conference on Artificial Intelligence, Virtual, 2–9 February 2021.

[16] Somepalli G., Goldblum M., Schwarzschild A., Bruss C.B., Goldstein T. (2021). SAINT: Improved Neural Networks for Tabular Data via Row Attention and Contrastive Pre-Training. arXiv.

[17] Huang X., Khetan A., Cvitkovic M., Karnin Z. (2020). TabTransformer: Tabular Data Modeling Using Contextual Embeddings. arXiv.

[18] Sakurada M., Yairi T. (2014). Anomaly Detection Using Autoencoders with Nonlinear Dimensionality Reduction. Proceedings of the MLSDA 2014 2nd Workshop on Machine Learning for Sensory Data Analysis, Gold Coast, QLD, Australia, 2 December 2014.

[19] An J., Cho S. (2015). Variational Autoencoder Based Anomaly Detection Using Reconstruction Probability.

[20] Zong B., Song Q., Min M.R., Cheng W., Lumezanu C., Cho D., Chen H. Deep Autoencoding Gaussian Mixture Model for Unsupervised Anomaly Detection. Proceedings of the 6th International Conference on Learning Representations.

[21] Ruff L., Kauffmann J.R., Vandermeulen R.A., Montavon G., Samek W., Kloft M., Dietterich T.G., Müller K.-R. (2021). A Unifying Review of Deep and Shallow Anomaly Detection. Proc. IEEE.

[22] Pang G., Shen C., Cao L., van den Hengel A. (2021). Deep Learning for Anomaly Detection: A Review. ACM Comput. Surv..

[23] Bahdanau D., Cho K., Bengio Y. Neural Machine Translation by Jointly Learning to Align and Translate. Proceedings of the 3rd International Conference on Learning Representations.

[24] Vaswani A., Shazeer N., Parmar N., Uszkoreit J., Jones L., Gomez A.N., Kaiser Ł., Polosukhin I. (2017). Attention Is All You Need. Advances in Neural Information Processing Systems 30.

[25] Woo S., Park J., Lee J.-Y., Kweon I.S. (2018). CBAM: Convolutional Block Attention Module. Proceedings of the European Conference on Computer Vision, Munich, Germany, 8–14 September 2018.

[26] Lin M., Chen Q., Yan S. Network in Network. Proceedings of the 2nd International Conference on Learning Representations.

[27] Le L., Patterson A., White M. (2018). Supervised Autoencoders: Improving Generalization Performance with Unsupervised Regularizers. Advances in Neural Information Processing Systems 31.

[28] Ghosh P., Sajjadi M.S.M., Vergari A., Black M.J., Schölkopf B. From Variational to Deterministic Autoencoders. Proceedings of the 8th International Conference on Learning Representations.

[29] Mondal A.K., Singhal L., Tiwary P., AP P., Singla P. (2023). Minority Oversampling for Imbalanced Data via Class-Preserving Regularized Auto-Encoders. Proceedings of the 26th International Conference on Artificial Intelligence and Statistics, Valencia, Spain, 25–27 April 2023.

[30] Tian M., Guo D., Cui Y., Pan X., Chen S. (2021). Improving Auto-Encoder Novelty Detection Using Channel Attention and Entropy Minimization. Proceedings of the 2nd ACM International Conference on Multimedia in Asia, Virtual, 7–9 March 2021.

[31] Zbontar J., Jing L., Misra I., LeCun Y., Deny S. (2021). Barlow Twins: Self-Supervised Learning via Redundancy Reduction. Proceedings of the 38th International Conference on Machine Learning, Virtual, 18–24 July 2021.

[32] Bardes A., Ponce J., LeCun Y. VICReg: Variance-Invariance-Covariance Regularization for Self-Supervised Learning. Proceedings of the 10th International Conference on Learning Representations.

[33] Bengio Y., Courville A., Vincent P. (2013). Representation Learning: A Review and New Perspectives. IEEE Trans. Pattern Anal. Mach. Intell..

[34] Ioffe S., Szegedy C. (2015). Batch Normalization: Accelerating Deep Network Training by Reducing Internal Covariate Shift. Proceedings of the 32nd International Conference on Machine Learning, Lille, France, 6–11 July 2015.

[35] Srivastava N., Hinton G., Krizhevsky A., Sutskever I., Salakhutdinov R. (2014). Dropout: A Simple Way to Prevent Neural Networks from Overfitting. J. Mach. Learn. Res..

[36] He K., Zhang X., Ren S., Sun J. (2016). Deep Residual Learning for Image Recognition. Proceedings of the IEEE Conference on Computer Vision and Pattern Recognition, Las Vegas, NV, USA, 27–30 June 2016.

[37] Loshchilov I., Hutter F. Decoupled Weight Decay Regularization. Proceedings of the 7th International Conference on Learning Representations.

[38] Kingma D.P., Ba J. Adam: A Method for Stochastic Optimization. Proceedings of the 3rd International Conference on Learning Representations.

[39] Glorot X., Bengio Y. (2010). Understanding the Difficulty of Training Deep Feedforward Neural Networks. Proceedings of the 13th International Conference on Artificial Intelligence and Statistics, Sardinia, Italy, 13–15 May 2010.

[40] Paszke A., Gross S., Massa F., Lerer A., Bradbury J., Chanan G., Killeen T., Lin Z., Gimelshein N., Antiga L. (2019). PyTorch: An Imperative Style, High-Performance Deep Learning Library. Advances in Neural Information Processing Systems 32.

[41] Pedregosa F., Varoquaux G., Gramfort A., Michel V., Thirion B., Grisel O., Blondel M., Prettenhofer P., Weiss R., Dubourg V. (2011). Scikit-Learn: Machine Learning in Python. J. Mach. Learn. Res..

[42] Chawla N.V., Bowyer K.W., Hall L.O., Kegelmeyer W.P. (2002). SMOTE: Synthetic Minority Over-Sampling Technique. J. Artif. Intell. Res..

[43] Cortes C., Vapnik V. (1995). Support-Vector Networks. Mach. Learn..

[44] Breiman L. (2001). Random Forests. Mach. Learn..

[45] van der Maaten L., Hinton G. (2008). Visualizing Data Using t-SNE. J. Mach. Learn. Res..

